# Vitamin C enhances co-localization of novel TET1 nuclear bodies with both Cajal and PML bodies in colorectal cancer cells

**DOI:** 10.1080/15592294.2024.2337142

**Published:** 2024-04-07

**Authors:** Nour El Osmani, Corinne Prévostel, Laurence Picque Lasorsa, Mohammad El Harakeh, Zeina Radwan, Hiba Mawlawi, Marwan El Sabban, Margret Shirinian, Zeina Dassouki

**Affiliations:** a IRCM, Institut de Recherche en Cancérologie de Montpellier, Montpellier, France; b Université de Montpellier, Montpellier, France; c Laboratory of Applied Biotechnology (LBA3B), AZM Center for Research in Biotechnology and its Applications, Doctoral School for Sciences and Technology, Tripoli, Lebanon; d INSERM, Montpellier, France; e ICM, Institut régional du Cancer de Montpellier, Montpellier, France; f Department of Anatomy, Cell Biology, and Physiological Sciences, Faculty of Medicine, American University of Beirut, Beirut, Lebanon; g Faculty of Public Health, Lebanese University, Tripoli, Lebanon; h Department of Experiment Pathology, Immunology, and Microbiology, American University of Beirut, Faculty of Medicine, Beirut, Lebanon; i Department of Medical Laboratory Sciences, University of Balamand, Faculty of Health Sciences, Tripoli, Lebanon

**Keywords:** TET1, nuclear bodies, vitamin C, Cajal bodies, PML bodies, partner proteins, CRC, 5hmC

## Abstract

Deregulation of ten-eleven Translocation protein 1 (TET1) is commonly reported to induce imbalances in gene expression and subsequently to colorectal cancer development (CRC). On the other hand, vitamin C (VitC) improves the prognosis of colorectal cancer by reprogramming the cancer epigenome and limiting chemotherapeutic drug resistance events. In this study, we aimed to characterize TET1-specific subcellular compartments and evaluate the effect of VitC on TET1 compartmentalization in colonic tumour cells. We demonstrated that TET1 is concentrated in coarse nuclear bodies (NB) and 5-hydroxymethylcytosine (5hmC) in foci in colorectal cancer cells (HCT116, Caco-2, and HT-29). To our knowledge, this is the first report of a novel intracellular localization profile of TET1 and its demethylation marker, 5hmC, in CRC cells. Interestingly, we found that TET1-NBs frequently interacted with Cajal bodies, but not with promyelocytic leukaemia (PML) bodies. In addition, we report that VitC treatment of HCT116 cells induces 5hmC foci biogenesis and triggers 5hmC marks to form active complexes with nuclear body components, including both Cajal and PML proteins. Our data highlight novel NB-concentrating TET1 in CRC cells and demonstrate that VitC modulates TET1-NBs’ interactions with other nuclear structures. These findings reveal novel TET1-dependent cellular functions and potentially provide new insights for CRC management.

## Introduction

Colorectal cancer (CRC) is the third most common cancer worldwide [1] and accounts for more than 10% of all cancer cases [1]. Among epigenetic processes, active DNA demethylation has been evidenced to be aberrantly regulated in the onset and/or progression of cancers [2]. The epigenetic regulator Ten-Eleven Translocation (TET) is a family of proteins that initiates demethylation by removing the epigenetic mark 5-methylcytosine (5mC) through a series of oxidation reactions [2]. Interestingly, an increasing number of alterations in the TET family have been found to induce imbalances in DNA demethylation, subsequently leading to cancer development [2,3]. Some studies have reported a tumour-suppressive role of TET in cancers [4]. Indeed, a significant increase in tumorigenesis was observed upon TET loss-of-function due to mutations or downregulation [5–7]. Conversely, other studies have reported that TET knockdown reduces cancer cell growth, highlighting the oncogenic properties of TET proteins [8,9].

According to literature, TET1 mRNA and protein expression levels are greatly decreased in CRC cells and tissues [10,11]. Consequently, the first oxidative product of the TET-induced active demethylation process, 5-hydroxymethylcytosine (5hmC), also showed a significant decrease [12]. In addition, studies indicate that TET1 decrease does not only promote CRC cell proliferation *in vitro* but also stimulates tumour volume and weight *in vivo* [13].

On the other hand, patients with CRC have low plasma vitamin C (VitC) levels compared to healthy individuals [14]. Recently, this antioxidant vitamin was reported to improve the quality of life of CRC patients by alleviating the side effects of chemotherapy [15]. According to Riordan *et al*, combined VitC chemotherapy treatment remarkably improved the survival outcome of patients with stage IV colon adenocarcinoma [16]. VitC was also shown to suppress tumour growth in several animal models and tissue culture studies [17]. Interestingly, several recent reports have revealed that VitC exerts its effect through the activation of TET proteins, as 5hmC levels remarkably increase after vitamin C treatment [17]. Although VitC is reported to upregulate the activity of TET proteins in an expression-independent manner, the molecular mechanism by which VitC activates TET1 is still not fully understood [18,19].

In this study, we identified a novel nuclear compartmentalization of TET1 protein in CRC cells compared to normal colon cells. Although the exact process of TET1/5hmC concentration in coarse nuclear dots is unknown, we assessed their interaction with common nuclear bodies and identified Cajal bodies as specific partner proteins of TET1-NBs in HCT116 cells. Furthermore, since Vitamin C has been reported to stimulate TET1-demethylation activity and restore 5hmC levels [17,20], we studied the impact of VitC treatment on TET1 activity. We observed VitC – mediated induction of promyelocytic leukaemia protein (PML) and Cajal bodies as well as their recruitment into active complexes with 5hmC. Thus, our results indicate a role for VitC in regulating the interdependent interplay between the nuclear bodies in cancer cells and suggest potential novel TET1-dependent cellular functions.

## Materials and methods

### Cell culture and treatment

The normal human colon epithelial cell line (NCM460) [21], cervical (HeLa), and colorectal adenocarcinoma cells (HCT116; Caco-2; HT-29) were obtained from Dr. Marwan El Sabban’s laboratory. HT-29 cells were cultured in RPMI-1640 medium (Sigma SR8758), whereas Caco-2, HCT116, and HeLa cells were maintained in DMEM medium (Sigma SD5796). Both media were supplemented with 10% Fetal Bovine Serum (FBS; Sigma SF9665) and 1% penicillin/streptomycin (P/S; Sigma L0022) solution at 37°C in a 5% CO2 humidified incubator. Cells were seeded in 24-well plates with coverslips for 24 h and then treated with 500 µM Vitamin C (VitC, Sigma 47,863) for 2 h. Culture media, FBS, P/S, and Vit C were purchased from Sigma-Aldrich, Lebanon.

### Immunofluorescence assay

Normal colon and CRC cell lines were fixed and permeabilized with ice-cold 100% methanol for at least 15 min at −20°C. Cells were blocked for 30 min in blocking buffer (0.25% BSA, 0.5% gelatin) and then incubated overnight with the following primary antibodies: rabbit anti-TET1 (1:500; Genetex GTX124207), rabbit anti-5hmC (1:500; Abcam ab214728 [RM236]), mouse anti-PML (1:500; Abcam ab96051), mouse anti-coilin (1:500; Abcam ab87913), SUMO1 (1:500; Santa Cruz Sc9060), and SUMO2/3 (1:500; Santa Cruz Sc32873). Cells were then incubated for 1 h with fluorochrome-labelled goat secondary antibodies: anti-rabbit Alexa Fluor 488 (1:500; Abcam ab150077) and/or anti-mouse Alexa Fluor 594 (1:500; Abcam ab150116). Nuclei were counterstained with DAPI (4,’6-diamidine-2-phenylindole dihydrochloride, Roche 10,236,276,001) dye and mounted with Prolong Gold anti-fade reagent (Invitrogen, P36930). Images were processed and analysed using confocal microscopy linked to the ZEN Imaging Software-LSM710. Post-confocal quantification and size measurement of the nuclear bodies were conducted using the ImageJ Software (NIH; https://imagej.nih.gov/ij/).

### Crystal violet assay

VitC cytotoxicity was assessed using a previously standardized crystal violet assay [22,23]. Briefly, colon cancer cells (5000 HCT116 and HT29 cells per well and 10,000 Caco-2 cells per well) were seeded in 24-well plates. Twenty-four hours later, the cells were incubated with VitC for 2 h at a final concentration of 500 µM. The cells were then stained with crystal violet (0.2% crystal violet; 2% ethanol) and lysed in 1% sodium dodecyl sulphate (SDS). The amount of deoxyribonucleic acid (DNA) intercalating agent, which is proportional to the number of cells, was measured at 570 nm with a PHERAstar® plate reader (BMG Labtech, Champigny Sur Marne, France).

### RNA extraction, reverse transcription, and quantitative real time PCR

Total RNA was extracted with 300 μl of TRIzol reagent. After 5 min, chloroform (60 μl) was added to each tube, mixed, and left for a few minutes, then centrifuged at 12,000 rpm for 15 minutes at 4°C. The colourless supernatant containing the RNA was then aspired and transferred to new tubes. Isopropanol (150 μl) was added and left at 4°C for 10 min, followed by centrifugation at 12,000 rpm for 15 min at 4°C, and the supernatant was discarded from the tubes. 150 μl of 75% ethanol was added to the remaining RNA pellet, followed by centrifugation at 7,500 rpm for 5 min at 4°C. The supernatant was discarded and the RNA pellet was then left to air-dry for 20 min at room temperature. 20 μl of nuclease-free water was then added to the pellet. Finally, RNA was quantified using a Nanodrop spectrophotometer (ND-1000 Spectrophotometer), and the samples were stored at −20°C.

2 μg of RNA will be reverse transcribed to cDNA using FIREScript® RT cDNA synthesis MIX (Solis Biodyne)

### Gene expression analysis

Quantitative real-time polymerase chain reaction (qRTPCR) was performed in a CFX96™ Real-Time PCR Detection System (Bio-Rad, Hercules, California, USA) using the qPCR Sybr Green Master Mix. The relative fold change in gene expression was calculated using the ΔΔ^Cq^ method after normalization to the housekeeping gene GAPDH. PCR settings was as follows: A pre-cycle at 95°C for 5 min followed by 40 cycles consisting of 95°C for 10 s, 60°C for 30 s, and 72°C for 30 s with a final extra-elongation at 72°C for 5 min.

Forward primer: 5’-GCTCTCATGGGTGTCCAATTGCT-3;’

Reverse primer: 5’-ATGAGCACCACCATCTCAGCAG-3’

### Statistical analysis

Statistical analysis was performed using STATA software for statistics and data science, and mean values are presented with standard deviation error bars. A one-way ANOVA statistical test was performed to compare the number and size of TET1/5hmC nuclear structures between normal colon and colorectal cancer cell lines. An independent Student’s t-test was used for comparative analysis of different nuclear body proteins, whereas a paired t-test was applied to assess NBs before and after VitC treatment. Pearson’s correlation coefficient (r) was used to analyse the correlations between variables. Differences were considered statistically significant at *p* < 0.05.

## Results

### Identification of novel TET1-nuclear bodies (TET1-NBs) in CRC cells

**Figure 1.**
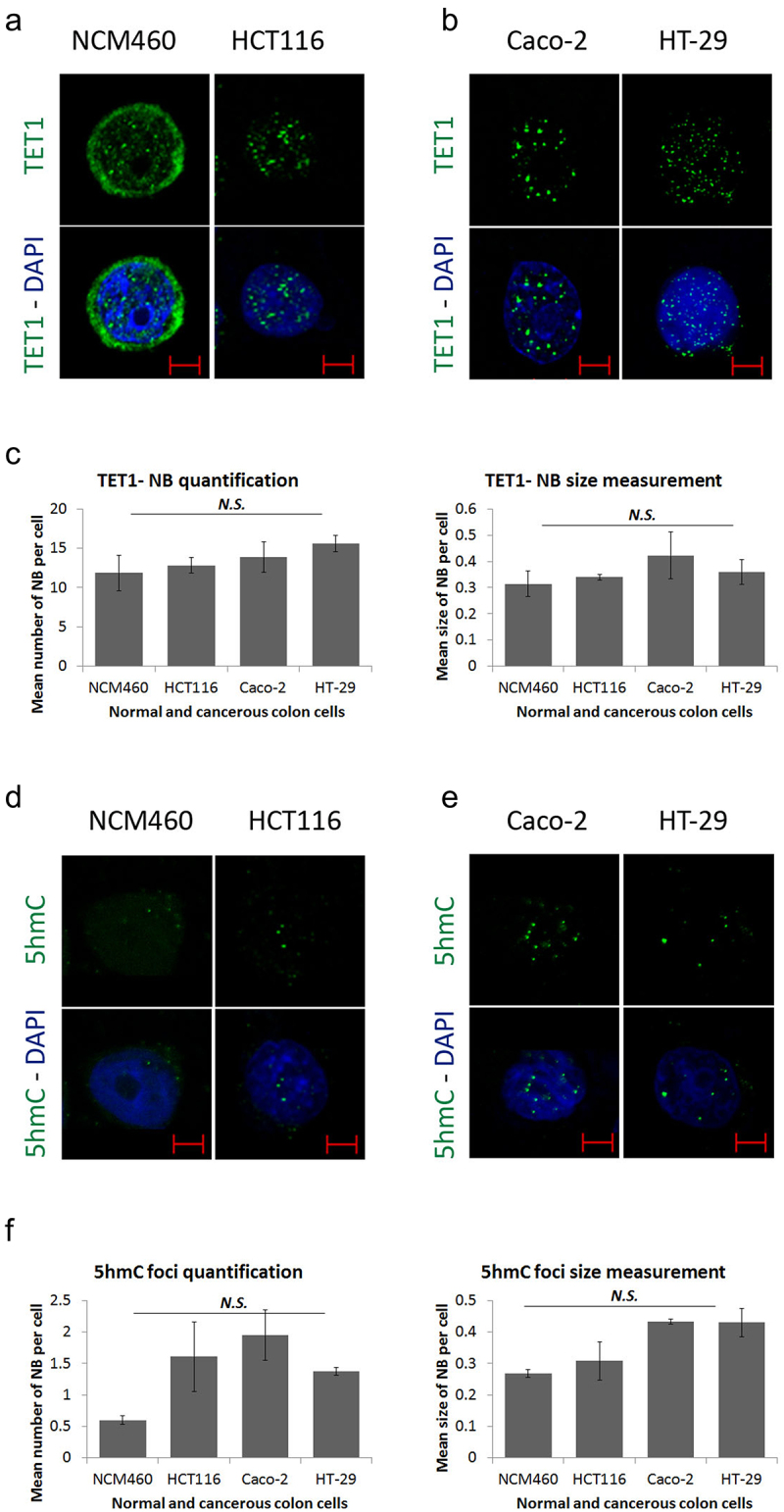
Formation of Ten-eleven translocation-nuclear bodies (TET1-NBs) and 5-hydroxymethylcytosine (5hmC) foci in CRC cells. Confocal images of (a) TET1 showing diffused distribution with few subtle nuclear structures as well as a cytoplasmic perinuclear profile in normal colon (NCM460) cells (n = 1). This intracellular distribution of TET1 concentrated in coarse nuclear bodies in HCT116 cells, (b) which is confirmed in other CRC cell lines (Caco-2, and HT-29) (n = 3). (c) For the comparative quantification and size measurement of TET1-NBs, we counted 50 cells from each cell line (NCM460, and CRC cells) (n = 3). (d) 5hmC, the demethylation mark of TET enzymes, displayed a comparable profile in NCM460 cells characterized by a randomly diffused distribution. However, 5hmC condensates into nuclear foci in (d) HCT116 cells, and in (e) Caco-2 and HT-29 cells. Nuclei are stained with DAPI (blue) with a scale bar: 5μm. (f) We counted 50 cells from NCM460 and from each CRC cell line for 5hmC foci.

To understand the role of TET1 in CRC, we assessed its intracellular localization in normal and colon cancer cell lines. Immunofluorescence showed random nuclear staining of TET1 along with a cytoplasmic perinuclear distribution in the normal colon epithelial cell line, NCM460 (Figure 1(a); Figure S1a). Strikingly, TET1 concentrated in coarse nuclear bodies (NBs), or ‘nuclear hotspots’ in HCT116 colon cells (Figure 1(a); Figure S1a). This profile was confirmed in two additional CRC cell lines (Caco-2, and HT-29) (Figure 1(b); Figure S1a). To further characterize these nuclear structures, TET1-NBs we quantified and measured the subtle versus coarse nuclear structures among the tested cell lines using ImageJ software. Results revealed that TET1-NBs reach up to 16 condensates per nucleus and range from 0.3 to 0.5 µm in size (Figure 1(c)). TET1-NBs number and size were not significantly different between NCM460 and different CRC cell lines (*p* = 0.805 and *p* = 0.388; for number and size, respectively) (Figure 1(c)). Next, we measured the expression level of TET1 gene across normal colon and CRC cells (Figure S1b). Using qRT-PCR, we showed that TET1 is highly expressed in HCT116 and Caco-2 cells compared to NCM460 and HT-29 counterparts (Figure S1b). These results suggest that the formation of TET1-NBs is independent of TET1 expression level.

Furthermore, we examined the distribution profile of 5hmC, a direct TET demethylation marker on DNA. In NCM460 cells, 5hmC staining was mainly diffused in the nucleus while completely concentrated to the nuclear foci in HCT116 cells (Figure 1(d); Figure S1c). The 5hmC foci were also observed in Caco-2 and HT-29 cells (Figure 1(e); Figure S1c). Post-confocal quantification and size measurement of foci showed no significant difference between the NCM460 and CRC cell lines (*p* = 0.385 and *p* = 0.466 for number and size, respectively) (Figure 1(f)). However, although TET1-NBs and 5hmC foci were similar in size, TET1-NBs were approximately 10-fold more numerous than 5hmC foci. Taken together, these data indicate a novel nuclear localization profile for TET1 and 5hmC in CRC cells.

### TET1-NBs are distinct from PML-NBs

**Figure 2.**
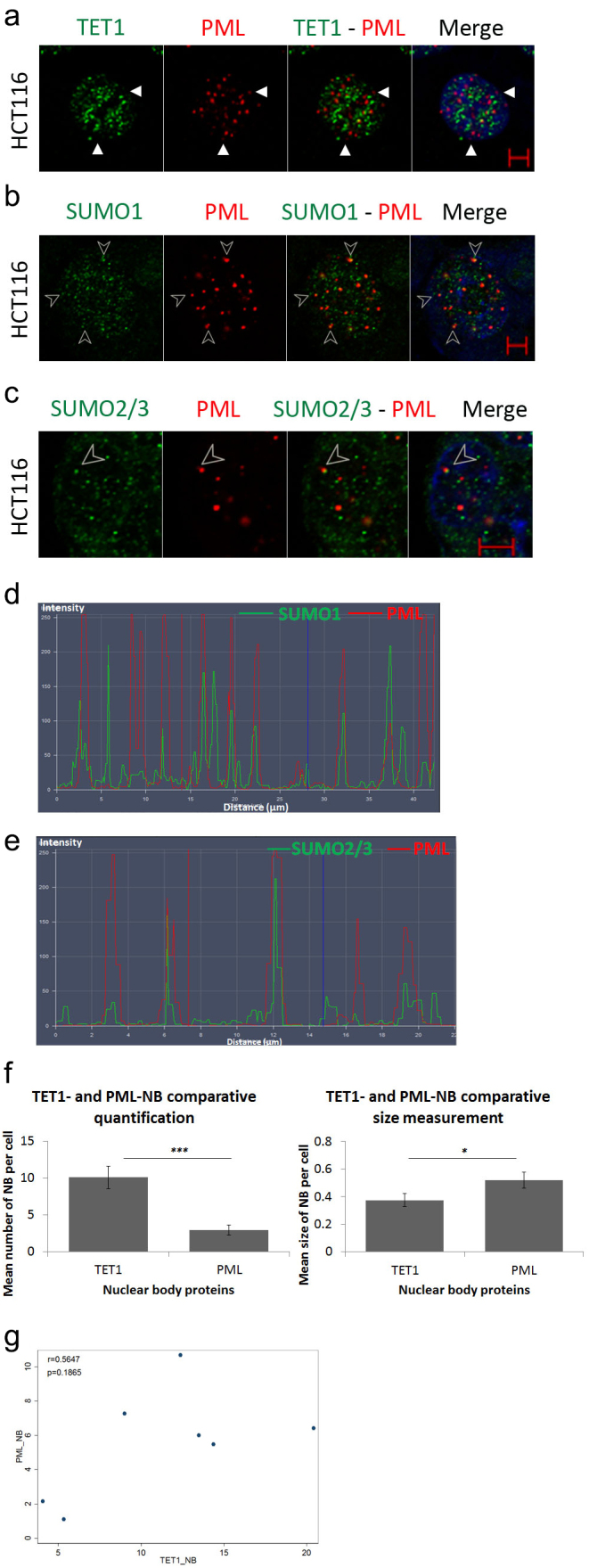
TET1 and promyelocytic leukemia protein (PML) are independent nuclear body proteins. (a) TET1 and PML show no co-localization as indicated by white arrowheads proteins in HCT116 cells (*n* = 3). To assess the ability of PML-NBs to recruit partner proteins, we co-immunostained PML-NBs with (b) SUMO1, and (c) SUMO2/3 (*n* = 3). The overlay of PML and SUMO isoforms is indicated by arrowheads and is confirmed by co-localization profile analysis in (d) and (e) (Scale bar: 5µm). (f) Comparative post-confocal analysis of count and size in 274 cells showing that PML-NBs are significantly fewer but larger in size with respect to the novel TET1-NBs in HCT116 cells. Error bars here represent the standard deviation of means (*p < 0.05; ***p < 0.001). (g) Pearson’s scatter plot shows no correlation between PML-and TET1-NBs in 562 cells (*r* = 0.6597; *p* = 0.5414).

Nuclear bodies are robust subnuclear structures that are actively involved in various molecular processes [24,25]. Promyelocytic Leukemia (PML) is a well-characterized tumour suppressor protein that recruits and orchestrates many proteins in PML-NBs [26]. Thus, we hypothesized that PML may confine TET1-NBs activity in CRC cells. To test this hypothesis, we performed a co-immunolabeling assay to compare the distribution of TET1- and PML-NBs in HCT116 cells. Confocal images revealed that the majority of TET1-NBs had localization independent of the master organizer PML protein (Figure 2(a); Figure S2(a)).

Therefore, we asked whether this outcome could reflect a defect in the ability of PML-NBs to recruit other partner proteins. To test this hypothesis, we assessed the intracellular profile of PML along with SUMO proteins, given that SUMOylation of PML is a prerequisite post-translational modification to confine target proteins in NB [27]. Indeed, SUMO1 and SUMO2/3 isoforms are indispensable components that favour the dynamic recruitment of various partner proteins into PML-NBs after the formation of the NB outer shell [27]. Confocal images displayed frequent co-localization of PML-NBs with SUMO1 and SUMO2/3 (Figure 2(b–)); Figure S2(b–c). In addition, a co-localization profile analysis validated the overlay of PML-NBs with SUMO1/2/3, suggesting that HCT116 cells might still contain a functional PML capable of recruiting other partner proteins (Figure 2(d–)); Figure S2(b–c).

Finally, comparative quantification of TET and PML nuclear body proteins indicated a significantly higher frequency of TET1-NBs (*p* = 0.0008) (Figure 2(f)). In addition, PML-NBs were significantly larger than TET1-NBs (*p* = 0.0286) (Figure 2(f)). Finally, Pearson’s statistical test indicated that the number of TET1 nuclear bodies showed no significant correlation with that of PML-NBs in HCT116 cells (562 cells; r = 0.6597; *p* = 0.5414; Figure 2(g)). Collectively, these observations suggested that TET1-NBs and PML bodies are independent nuclear hotspots.

### TET1-NBs co-localize with Cajal bodies

**Figure 3.**
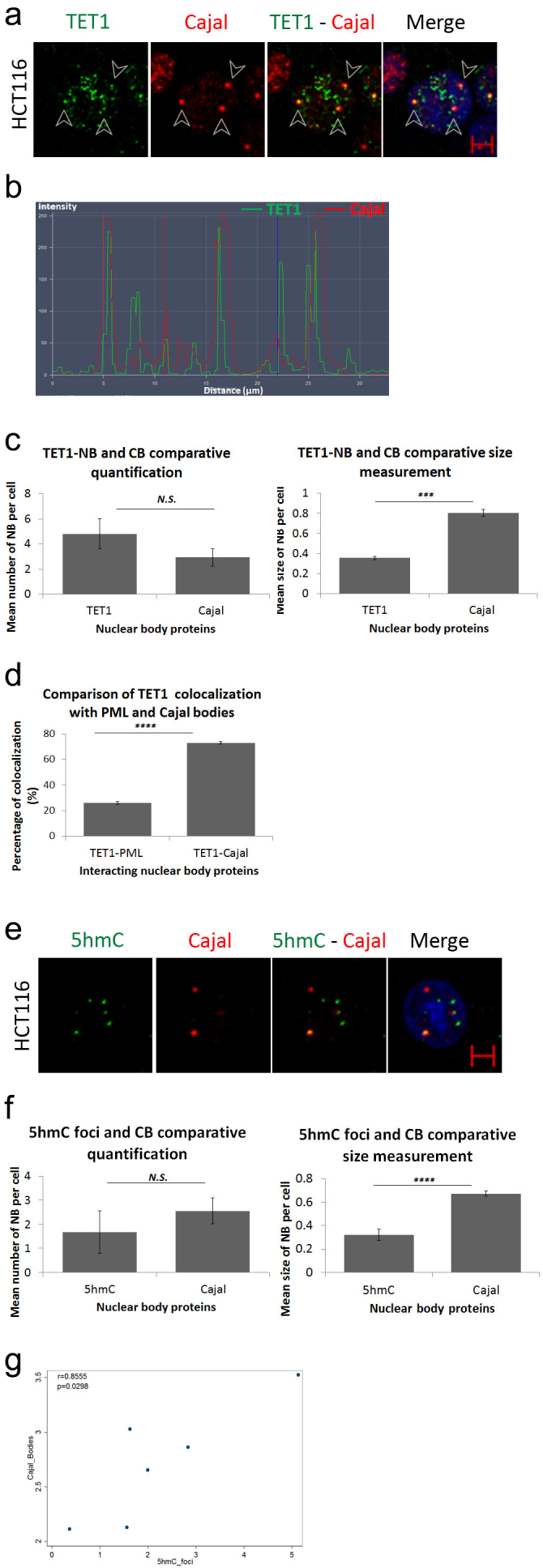
TET1 and its demethylation mark, 5hmC, interact with Cajal bodies. (a) Confocal images showing co-localization of TET1-NBs and CBs in HCT116 cells as indicated by arrowheads (*n*=3; Scale bar: 5µm). (b) Co-localization profile analysis of nuclear TET1-NBs and CBs further demonstrates the overlay of the two structures. (c) Comparative quantification and size measurement of TET1-NBs and CBs in 308 cells. (d) Comparative analysis showing significant difference between the co-localization percentage of TET1-PML and TET1-Cajal. (e) Similarly, the direct demethylation mark, 5hmC, also co-localized with CBs. (f) Post-confocal analysis in 241 cells showed that 5hmC foci were not significantly fewer than Cajal bodies while, as observed for TET1-NBs, 5hmC foci were significantly smaller than CBs. Error bars represent the standard deviation of means (***p < 0.001; ****p < 0.0001; N.S: non-significant) (g) Pearson’s scatter plot shows a positive correlation between Cajal bodies and 5hmC foci in 264 cells (*r* = 0.8555; *p* = 0.0298).

Since TET1-NBs are distinct from PML-NBs, we examined other prominent nuclear structures that could potentially characterize TET1-NBs. For this purpose, we selected Cajal Bodies (CBs), another prototypical nuclear organelle involved in RNA processing [28]. We targeted coilin-p80, a common marker protein of CBs, along with TET1, in HCT116 cells. Interestingly, 73% of the coilin *p*-80 positive NBs co-localized with the TET1 protein (Figure 3(a); Figure S3.A). Analysis of the co-localization profile further confirmed the overlay of nuclear TET1 and CBs (Figure 3(b)). Post-confocal analysis revealed no significant difference between the numbers of TET1-NBs and CBs (*p* = 0.0969). Since TET1-NBs were significantly smaller than CBs (*p* = 0.0003) (Figure 3(c)), TET1-NBs are most likely partners of Cajal bodies rather than identical nuclear structures. However, the statistically significant difference between the co-localization percentage of TET1-PML (26%) and TET1-Cajal (73%) suggests that Cajal bodies are frequent nuclear partners of TET1-NBs (*p* = 0.00001; Figure 3(d)). Moreover, 16% of TET1-NBs positive cells co-localize with total CBs, whereas 12% of CB-positive cells appeared to co-localize with TET1 in HCT116 cells, suggesting a predominant function of TET1 in these cells (Supplementary Table S1).

To assess whether 5hmC is also concentrated in CBs, we performed immunostaining for coilin-p80 along with 5hmC in HCT116 cells. As observed for TET1, 5hmC colocalized with CBs (Figure 3(e); Figure S3b). Again, CBs were significantly greater in size with respect to 5hmC foci (*p* = 0.00001) (Figure 3(f)). We also assess the percentage of nuclear structures enriched for 5hmC and Cajal bodies positive staining based on total counts of either CBs or 5hmC foci. This showed that up to 12% of nuclear structures display positive enrichment of both staining marks and that this percentage increased to 20% after VitC treatment. The increase in the percentage of 5hmC-and CB-positive nuclear structures from 11% to 20% with VitC treatment suggests that VitC potentiates the co-localization of 5hmC and CBs in HCT116 cells (Supplementary Table S1). Moreover, we conducted Pearson’s correlation analysis to explore the relationship between nuclear structure quantification of 5hmC foci and CB nuclear bodies. The analysis revealed a significant positive correlation between the number of each nuclear structure (5hmC and CBs) in HCT116 cells (264 cells; r = 0.8555; *p* = 0.0298; Figure 3(g)). To our knowledge, this is the first evidence of an association between 5hmC levels and CBs.

### VitC favors demethylation, promotes NB biogenesis, and enhances the association of 5hmC with partner proteins

**Figure 4.**
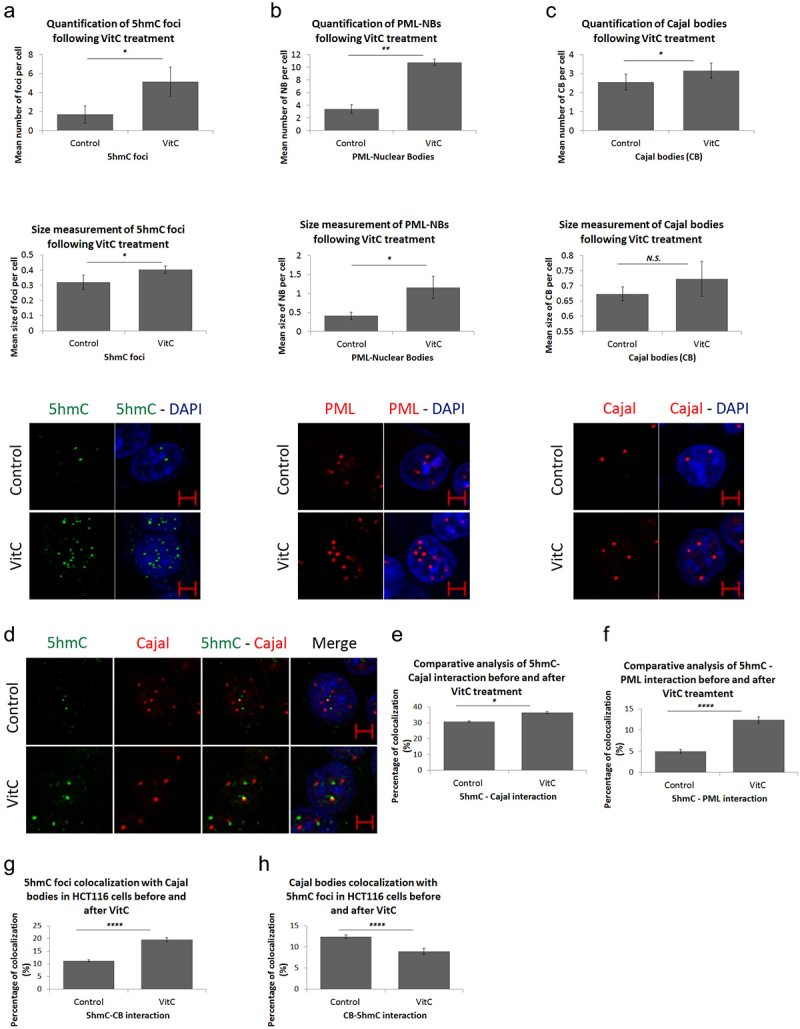
Vitamin C promotes nuclear body biogenesis and enhances co-localization in HCT116 cells. Comparative confocal and post-confocal analysis showing a significant increase, upon VitC treatment, in the number and size of (a) 5hmC foci in 241 cells, (b) PML-NBs in 152 cells, and (c) CBs in 241 cells (*n* = 3). (d) Immunofluorescence images showing the intracellular distribution pattern of 5hmC foci and Cajal bodies following 500 µM of Vitamin C (*n* = 3; Scale bar: 5µm). VitC treatment significantly increased the co-localization of 5hmC with PML-NBs and Cajal bodies in (e) and (f) respectively. (g) the co-localization percentage of 5hmC foci with CBs increased by 9% upon VitC treatment however, in (h) the inverse co-localization percentage showed a 3% decrease in CBs co-localization with 5hmC foci in 195 cells. Error bars here represent the standard deviation of means (*p < 0.05; **p < 0.01; ****p < 0.0001; N.S: non-significant).

In addition to its clinical use as an adjuvant in the treatment of various types of cancer, VitC greatly induces the demethylation activity of TET proteins [15,17]. First, we investigated whether exposing CRC cells to a pharmacological dose of VitC would affect their viability. Using the crystal violet assay, we observed that 500 µM VitC did not induce any significant cytotoxicity to CRC cells (Figure S4). Next, we assessed how these novel nuclear structures responded to VitC exposure. Immunostaining and post-confocal analysis revealed a significant increase in the number and size of 5hmC foci upon VitC treatment (*p* = 0.0135 and *p* = 0.0357, respectively) (Figure 4(a)), suggesting that VitC stimulates the formation of 5hmC foci in HCT116 cells.

To address the question of whether the effects of VitC are restricted to 5hmC foci, we examined the impact of VitC on other nuclear body proteins, including PML-NBs and CBs. A significant increase in PML-NBs (*p* = 0.0099 and *p* = 0.0445) and number of CBs (*p* = 0.0352) was observed in HCT116 cells upon VitC exposure (Figure 4(b–)). This increase was further observed in Caco-2 and HT-29 cells (Figure S5), suggesting that VitC could be a general inducer of biogenesis of nuclear structures.

Since VitC induced both 5hmC and CBs, we suspected that VitC might enhance the interaction between both structures. Upon treatment with VitC, the percentage of 5hmC-Cajal co-localization significantly increased from 31% to 36% (*p* = 0.0142; Figure 4(d–)); Fig.S6). Similarly, VitC significantly enhanced the 5hmC-PML overlap by 7% in HCT116 cells (*p* = 0.00001; Figure 4(f) ; Fig.S6). Hence, VitC stimulated the association of 5hmC with PML and CBs. Moreover, to identify which nuclear structure functionally predominates in HCT116 cells, we assessed the co-localization percentage of 5hmC foci with Cajal bodies and vice versa. The percentage of 5hmC foci-positive cells colocalized with total CBs significantly increased from 11% to 20% upon Vitamin C treatment (*p* < 0.0001). On the other percentage of CB-positive cells co-localization percentage with total 5hmC foci significantly decreased by 3% with treatment (*p* < 0.0001), which might be explained by the higher VitC – mediated induction of 5hmC foci with respect to CBs (Figure 4(g,h); Supplementary Table S1). Together, our data suggest that VitC has multiple effects on both the biogenesis and dynamics of intra-nuclear organelles in CRC cells.

## Discussion

In the present study, we report that TET1 is distributed into subtle nuclear structures that are randomly diffused in the nucleoplasm along with a perinuclear distribution in normal epithelial colon mucosa (NCM460) cells. This profile concentrates in nucleoplasmic coarse nuclear bodies (TET1-NBs) in CRC cells, indicating that TET1 is a newly characterized nuclear body protein in CRC (Figure 1; Figure S1). We observed a similar profile when immunolabeling cells with TET2 and TET3, suggesting that these novel nuclear are not spontaneously formed (Fig. S7). Over the past decade, few studies have shown the nuclear diffusion of the TET1 protein in normal mammalian cell lines, particularly in human embryonic kidney (HEK239T) and mammary epithelial (MCF-12a) cells [29–31]. Although it is localized to the nucleus at baseline conditions, our unprecedented finding of a TET1 cytoplasmic perinuclear profile in NCM460 cells is intriguing. According to Arioka et al., changes in the expression levels of activation-induced cytidine deaminase (AID) enzymes regulate the intracellular localization of TET1 [31]. Namely, AID promoted cytoplasmic translocation of TET1 in HEK239T cells [31]. Another study by Wu et al. showed that cells lacking retinoic acid receptor beta (RARβ) displayed cytoplasmic TET2, while RARβ re-expression restored nuclear TET2 compartmentation [29]. Moreover, O2 also influence TET1 localization in mouse trophoblast stem cells [32], indicating that multiple intra- and extracellular factors may influence the TET1 profile.

Besides TET-interacting factors (e.g., AID and RARβ), O-linked β-N-acetylglucosamine (OGT)-mediated post-translational modification appeared to export TET3 and disrupt 5hmC formation in HEK239T cells [33]. Moreover, TET3, which displays a minor cytoplasmic distribution, may be conveyed directly from the oocyte cytoplasm to the paternal nucleus after fertilization [3]. On the other hand, TET1 and TET2 are expelled from the nucleoplasm via their nuclear export signals in gastric cancer and small intestinal neuroendocrine tumour cells, respectively [34,35]. Using a series of green fluorescent protein-tagged and mutant constructs, Xiao et al.. (2013) identified a conserved nuclear localization signal (KKRK) in mouse TET1/3, indicating that the nuclear localization and/or translocation of these proteins into the nuclear compartment might be favoured by importin-α/β [36]. Finally, abnormal nuclear translocation of TET2 from the cytosol has recently been reported in a non-tumoural but chronic stress situation through an Abelson helper integration site-1 (Ahi1), a dependent process in neuronal cells [37]. These findings suggest several nuclear transport mechanisms involving nuclear export/import signals, partner proteins, and/or post-translational modifications [29,31,33,35–37]. The prominent cytoplasmic TET1 detected in NCM460 cells may point towards TET1 forming a reserve pool bridging different cellular compartments to cope with the increasing need for global hypomethylation during carcinogenesis and/or trigger TET1 catalytic-dependent and -independent functions throughout malignant cell transformation. This issue, which is by itself the subject of this article, will have to be addressed in the future.

Given that our results indicate the concentration of diffused 5hmC profile into coarse nuclear foci in CRC cells (Figure 1; Figure S1), it remains quite possible that catalytic-inactivation mutations are absent or that TET paralogs also actively drive this profile of the 5hmC mark. We suspect that the recruitment of TET1 and 5hmC into nuclear structures may implicate a novel compensatory mechanism to activate TET-mediated demethylation in CRC. However, further studies are required to validate this hypothesis.

NBs are robust structures that confine many components involved in various mechanisms in the nucleoplasm [24,25]. It is believed that the interactions and processes within the restricted space of NBs are of higher efficiency and have a higher speed rate [24,38]. Since TET1-NBs have never been described, we were intrigued to identify whether these nuclear hotspots interact with the most commonly studied nuclear structures, such as Promyelocytic Leukemia nuclear bodies (PML-NBs) and/or Cajal bodies (CB).

PML self-assembles into discrete nuclear structures in a process mediated by the recruitment of many partner proteins (as SP100, DAXX, and SUMO1/2/3 [39]. The ubiquitous PML-NBs sequester and release a wide range of substrates in order to regulate several crucial cellular processes (DNA repair, tumour suppression, apoptosis, antiviral responses, etc…) [26,40]. Although PML is the master organizer of an ever-expanding number of proteins, we revealed a great distinction between TET1- and PML-NBs (Figure 2; Figure S2). This outcome suggests NB-specific cellular functions in HCT116 cells.

**Figure 5.**
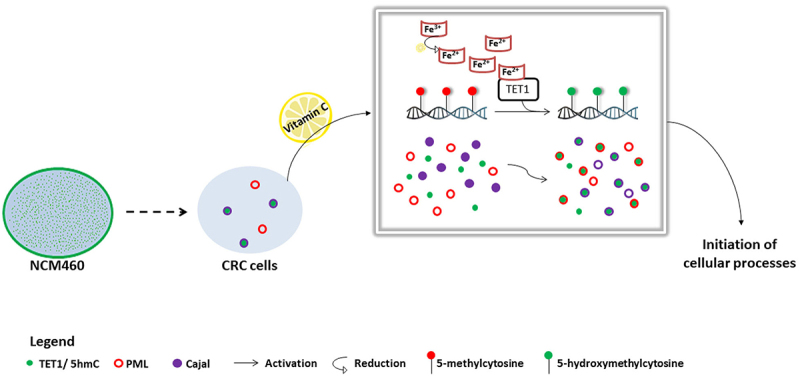
Proposed model of TET1 and 5hmC activation in HCT116 cells. The intracellular profile of TET1 and 5hmC shifted from a diffused distribution in colon cells (NCM460) to the formation of coarse nuclear structures in CRC cells. Our data reveal the particular association between TET1 and 5hmC nuclear condensates and Cajal bodies. VitC-mediated activation of TET1 promoted both the biogenesis of nuclear structures as well as the interaction of 5hmC with nuclear body proteins (i.e., PML and Cajal). VitC promotes the re-localization, at least in part, of 5hmC to these nuclear structures. These findings suggest the implication of TET1 in initiating several cellular mechanisms (i.e., possibly modifying RNA enriched in Cajal bodies).

CBs are distinct, frequently studied dynamic nuclear structures that continuously exchange components with the nucleoli [25,41]. Here, our results showed a TET1-Cajal co-localization of 73% and (5hmC-Cajal displayed a significant positive correlation in HCT116 cells (Figure 3; Figure S3). These findings indicate that TET1 could constitute a frequent functional partner of CBs and potentially play a role in RNA biogenesis and/or processing. Indeed, Fu et al. revealed that TET-mediated demethylation is not limited to DNA, and that TET proteins are capable of efficiently hydroxymethylating 5mC in RNA to 5hmC both in vitro and in vivo [42]. In *Drosophila melanogaster*, a single TET protein, dTET, induces demethylation of 5mC on both DNA (m6A) and RNA (5mrC) [43,44]. Another set of studies has disclosed the implication of the 5hmC mark in regulating the stability of mRNA in mouse embryonic stem cells (mESC) [45–47]. The diminished translational efficiency of oxidized mRNA transcripts is subsequent to their shorter half-life and faster degradation [45–47]. The destabilization effects of 5hmC demethylation marks have also been applied to non-coding RNA (ncRNAs). According to He et al., transfer RNA, – evidenced as a major target of TET2 – is greatly regulated in terms of biogenesis and expression levels in mESC [46]. Moreover, it is worth noting that cytosine methylation of non-coding microRNAs contributes to the loss of their repressive functions [48]. Subsequently, miRNA methylation inhibits the formation of duplexes with mRNA transcripts to suppress their translation [48]. This discovery sheds light on the epigenetic regulation of non-coding RNA. Given that small nuclear ribonucleoproteins (snRNPs) and small nucleolar ribonucleoproteins (snoRNPs) are processed in Cajal bodies, a synergistic interaction between other ncRNAs and CBs was recently identified. In fact, the biogenesis and maturation of microRNAs appear to take place in CBs [49]. The disruption of Cajal bodies by knocking out one of its major constituents significantly reduced miRNA levels in human choriocarcinoma cells [49]. Additionally, ncRNA activity is deregulated by aberrant DNA hypermethylation [50]. Collectively, these observations, together with our findings could indicate a link between TET epigenetic functions and RNA-mediated regulation of gene expression. Indeed, we suspect that TET1-Cajal co-localization might be an indispensable step towards the activation of non-coding RNA to control cellular processes. In Figure 5, we present a model summarizing our results and speculating on possible downstream effects mediated by the TET1-Cajal interaction (Figure 5). The next step is to examine the molecular basis of this co-localization and evaluate its effects on RNA modulation and its possible involvement in cancer progression.

Our analysis of TET1-Cajal interdependent percentage of co-localization in HCT116 cells revealed the functional predominance of TET1 over CBs (Table S1). These percentages were not assessed following VitC treatment since VitC upregulates the catalytic activity of TET proteins in an expression-independent manner [18,19]. In fact, it is not surprising to detect TET1-devoided CBs, as CBs have been implicated in multiple RNA-related metabolic processes, including non-coding RNA maturation and telomere maintenance [28,49]. Thus, it is expected that CBs may have TET-independent roles. Furthermore, CBs may also possibly recruit different TET proteins depending on RNA processing. All of these hypotheses should be investigated in future studies to elucidate the consequences of TET1 interactions with CBs.

Finally, NBs proteins dynamically respond to external modulators, particularly to chemotherapeutic drugs. For instance, arsenic has been reported to stimulate the biogenesis of PML-NBs following the degradation of PML-RARα fusion oncoprotein in acute promyelocytic leukaemia cells [51]. Similarly, doxorubicin treatment drives the formation of PML-NBs and triggers the direct interaction between PML and TET2 [52]. As both drugs and VitC modulate oxidative stress to exert their effects, we studied the impact of VitC on different NB proteins, including the newly characterized 5hmc foci. Our results demonstrated that VitC significantly promoted the formation of PML- and Cajal-NBs in CRC cells (Figure 4; Figure S5). Besides, VitC does not only increase 5hmC foci in terms of size and number but also favours the recruitment of 5hmC into PML- and Cajal-NBs (Figure 4; Fig.S6). Assessment of the 5hmC-Cajal interdependent co – localization percentage in the presence of VitC revealed the functional predominance of 5hmC over its nuclear counterpart (CBs) in HCT116 cells (Figure 4; Table S1), suggesting that VitC-induced TET1 activation mediates the formation of active 5hmC complexes with PML and Cajal nuclear body proteins.

Taken together, our findings evidence that VitC promotes the biogenesis of NBs and enhances the interactions among distinct nuclear components. Whether the TET1-Cajal interaction plays a role in RNA regulation remains a hypothesis that should be further validated (Figure 5).

In conclusion, we report the shift of TET1 and 5hmC from a diffused nuclear distribution in control NCM460 cells to the formation of TET1-nuclear bodies and 5hmC foci in CRC cells. Based on this novel profile, we believe that our observations are a starting point for further studies to identify possible new functions of TET1, including its potential impact on post-transcriptional RNA modifications.

## Supplementary Material

-) Supplementary files.docx

## Data Availability

All data generated or analysed during this study are included in this published article and its supplementary files.
