# Supplementary material for: Vitamin C enhances co-localization of novel TET1 nuclear bodies with both Cajal and PML bodies in colorectal cancer cells
Source: Epigenetics. 2024 Apr 7;19(1):2337142. doi: 10.1080/15592294.2024.2337142 (PMC11000620; doi:10.1080/15592294.2024.2337142)
Supplement: -) Supplementary files.docx [file KEPI_A_2337142_SM3547.docx]

Vitamin C enhances co-localization of novel TET1 nuclear bodies with both Cajal and PML bodies in colorectal cancer cells

Nour El Osmani ^1,2,3†^, Corinne Prévostel ^1,2,4,5*†,^ Laurence Picque Lasorsa ^1,2,4,5^, Mohammad El Harakeh ^6^, Zeina Radwan ^6^, Hiba Mawlawi ^3,7^, Marwan El Sabban ^6^, Margret Shirinian ^8 ‡^and Zeina Dassouki ^3,9 *‡^

1. IRCM, Institut de Recherche en Cancérologie de Montpellier, Montpellier, France
2. Université de Montpellier, Montpellier, France
3. Laboratory of Applied Biotechnology (LBA3B), AZM Center for Research in Biotechnology and its Applications, Doctoral School for Sciences and Technology, Lebanon
4. INSERM, U1194, Montpellier, France
5. ICM, Institut régional du Cancer de Montpellier, Montpellier, France
6. Department of Anatomy, Cell Biology, and Physiological Sciences, Faculty of Medicine, American University of Beirut, Lebanon
7. Faculty of Public Health, Lebanese University, Tripoli, Lebanon
8. Department of Experiment Pathology, Immunology, and Microbiology, American University of Beirut, Faculty of Medicine, Beirut, Lebanon
9. Department of Medical Laboratory Sciences, University of Balamand, Faculty of Health Sciences, Lebanon

† Co-first authors equally contributed; ‡Co-last authors equally contributed

* Correspondence: corinne.prevostel@inserm.fr (C.P.); ([zeina.dassouki@balamand.edu.lb](mailto:zeina.dassouki@balamand.edu.lb) (Z.D.)

## Supplementary figures


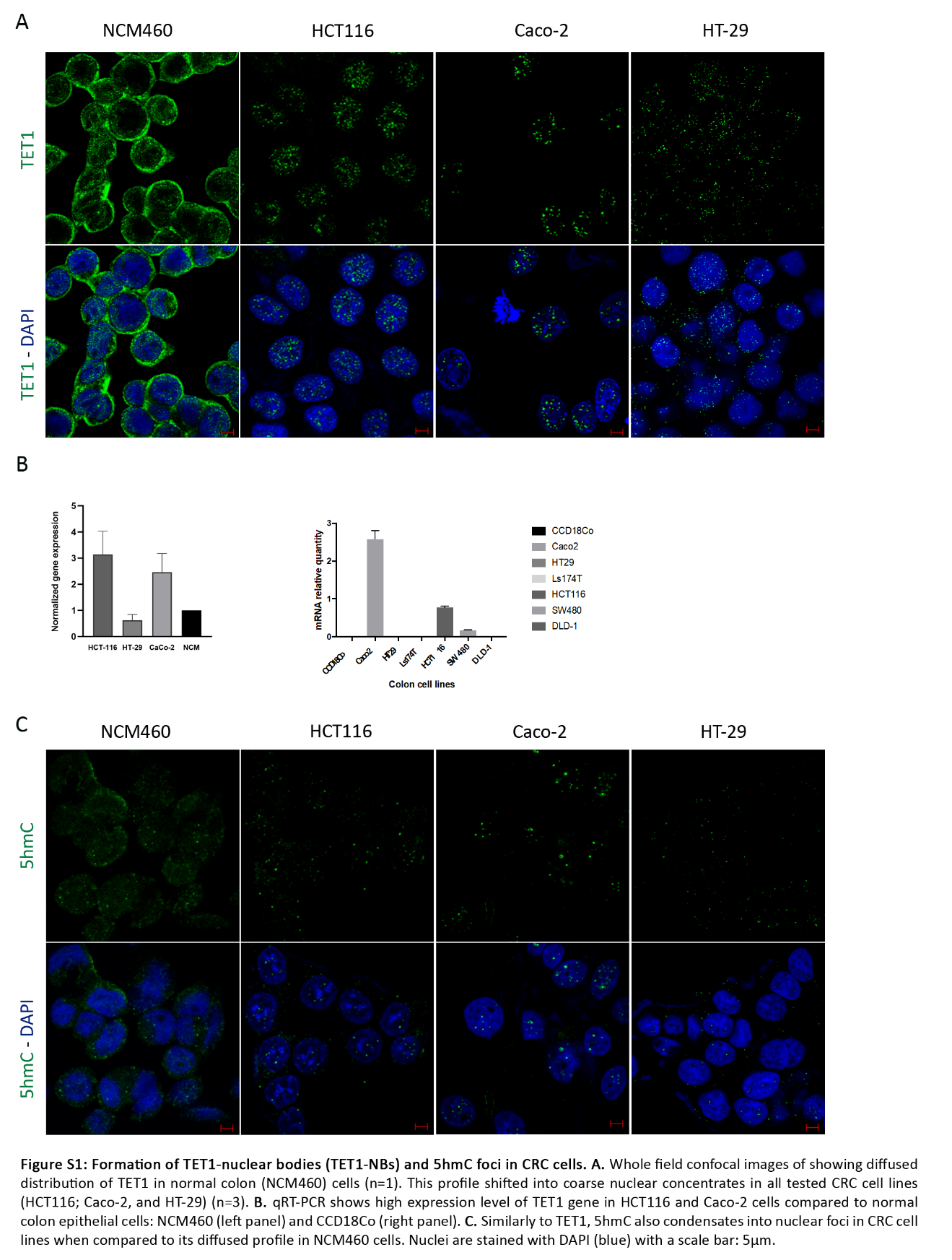


**Figure S1: Formation of TET1-nuclear bodies (TET1-NBs) and 5hmC foci in CRC cells**. **A.** Whole field confocal images of showing diffused distribution of TET1 in normal colon (NCM460) cells (n=1). This profile shifted into coarse nuclear concentrates in all tested CRC cell lines (HCT116; Caco-2, and HT-29) (n=3). **B.** qRT-PCR shows a high expression level of TET1 gene in HCT116 and Caco-2 cells compared to normal colon epithelial cells: NCM460 (left panel) and CCD18Co (right panel). **C.** Similarly to TET1, 5hmC also condensates into nuclear foci in CRC cell lines when compared to its diffused profile in NCM460 cells. Nuclei are stained with DAPI (blue) with a scale bar: 5μm.

**
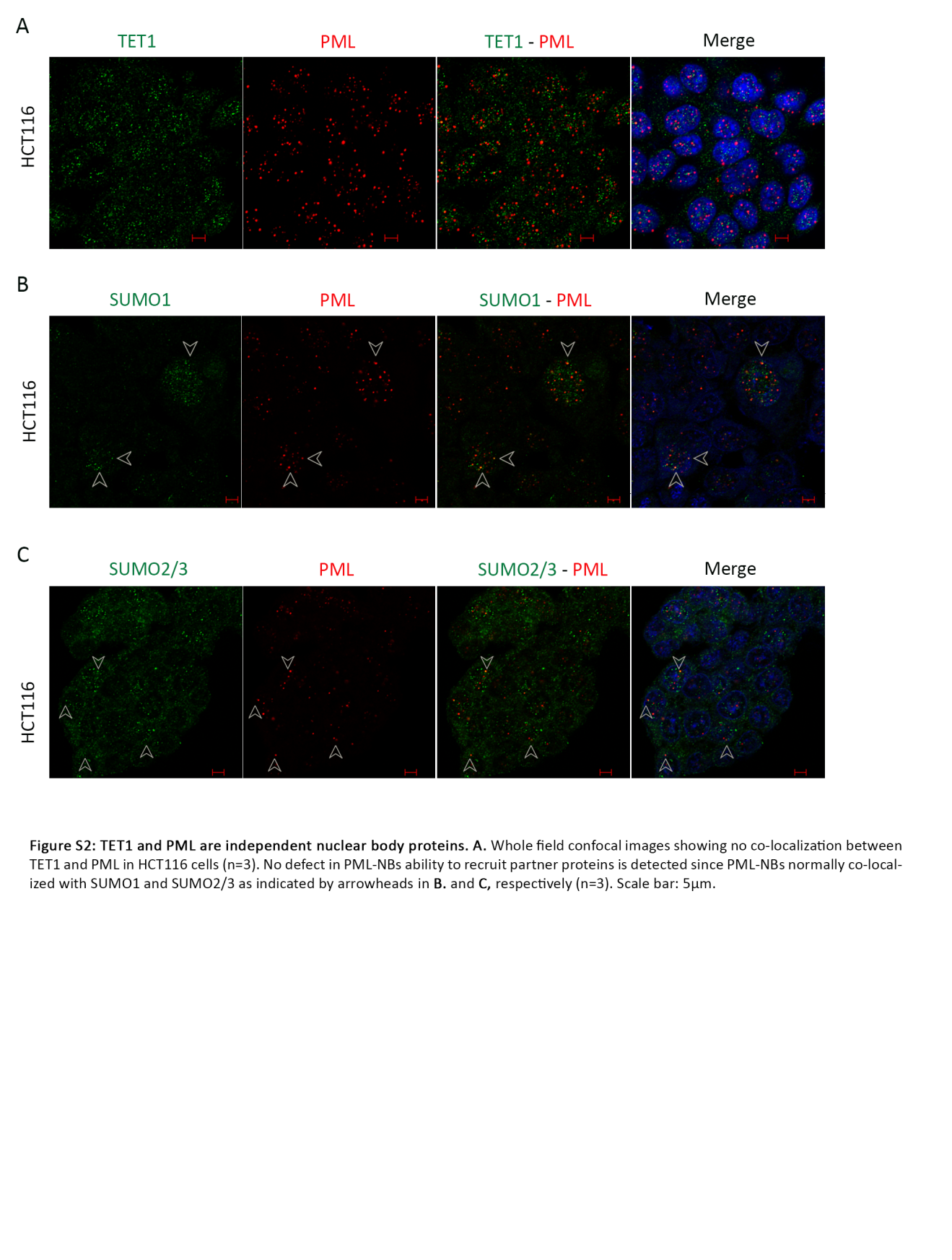
**

**Figure S2: TET1 and PML are independent nuclear body proteins. A.** Whole field confocal images showing no co-localization between TET1 and PML in HCT116 cells (n=3). No defect in PML-NBs ability to recruit partner proteins is detected since PML-NBs normally co-localized with SUMO1 and SUMO2/3 as indicated by arrowheads in **B.** and **C**, respectively (n=3). Scale bar: 5µm.

**
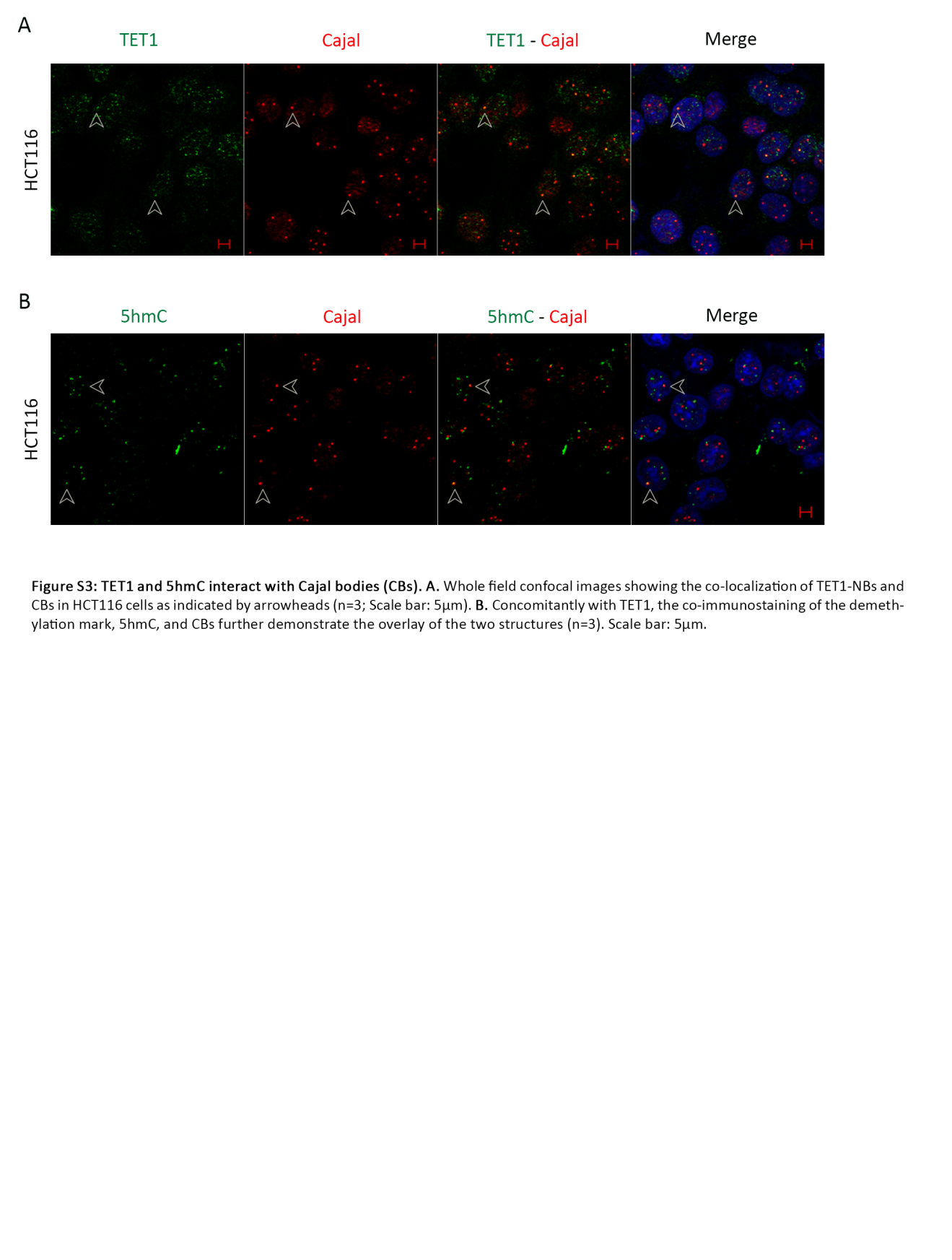
**

**Figure S3: TET1 and 5hmC interact with Cajal bodies (CBs). A.** Whole field confocal images showing the co-localization of TET1-NBs and CBs in HCT116 cells as indicated by arrowheads (n=3; Scale bar: 5µm). **B.** Concomitantly with TET1, the co-immunostaining of the demethylation mark, 5hmC, and CBs further demonstrate the overlay of the two structures (n=3). Scale bar: 5µm.


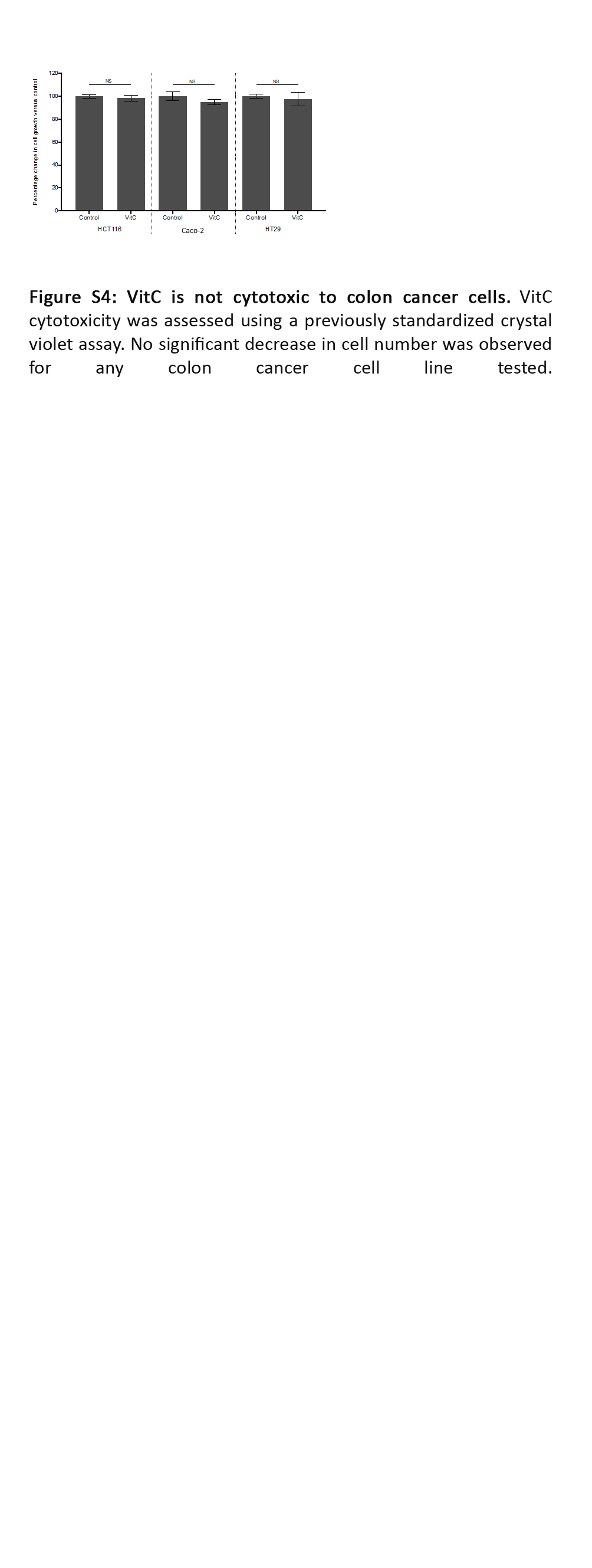


**Figure S4: A 2-hour exposure to VitC showed no cytotoxicity in colon cancer cells.** VitC cytotoxicity was assessed using a previously standardized crystal violet assay. No significant decrease in cell number was observed for any colon cancer cell line tested.


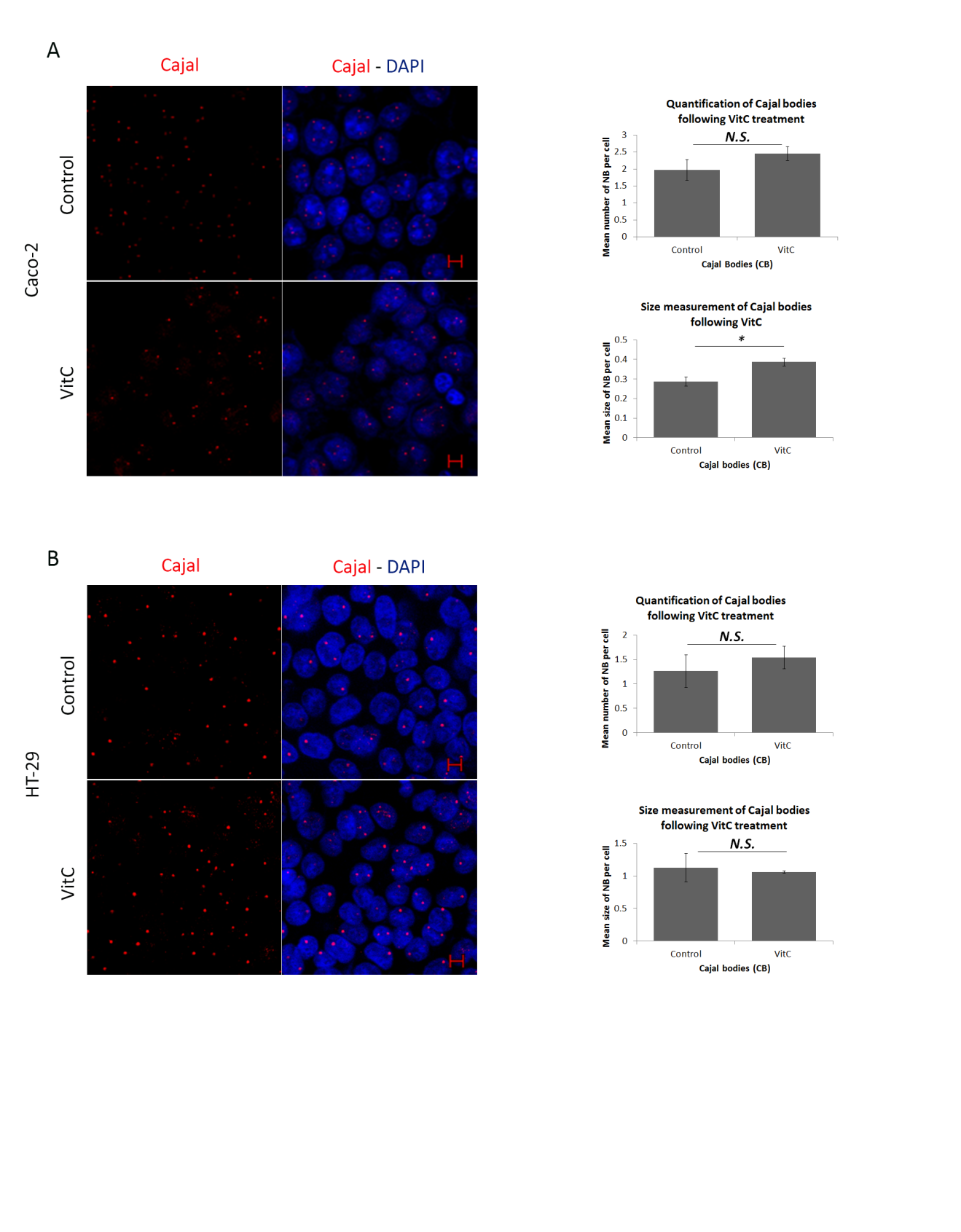


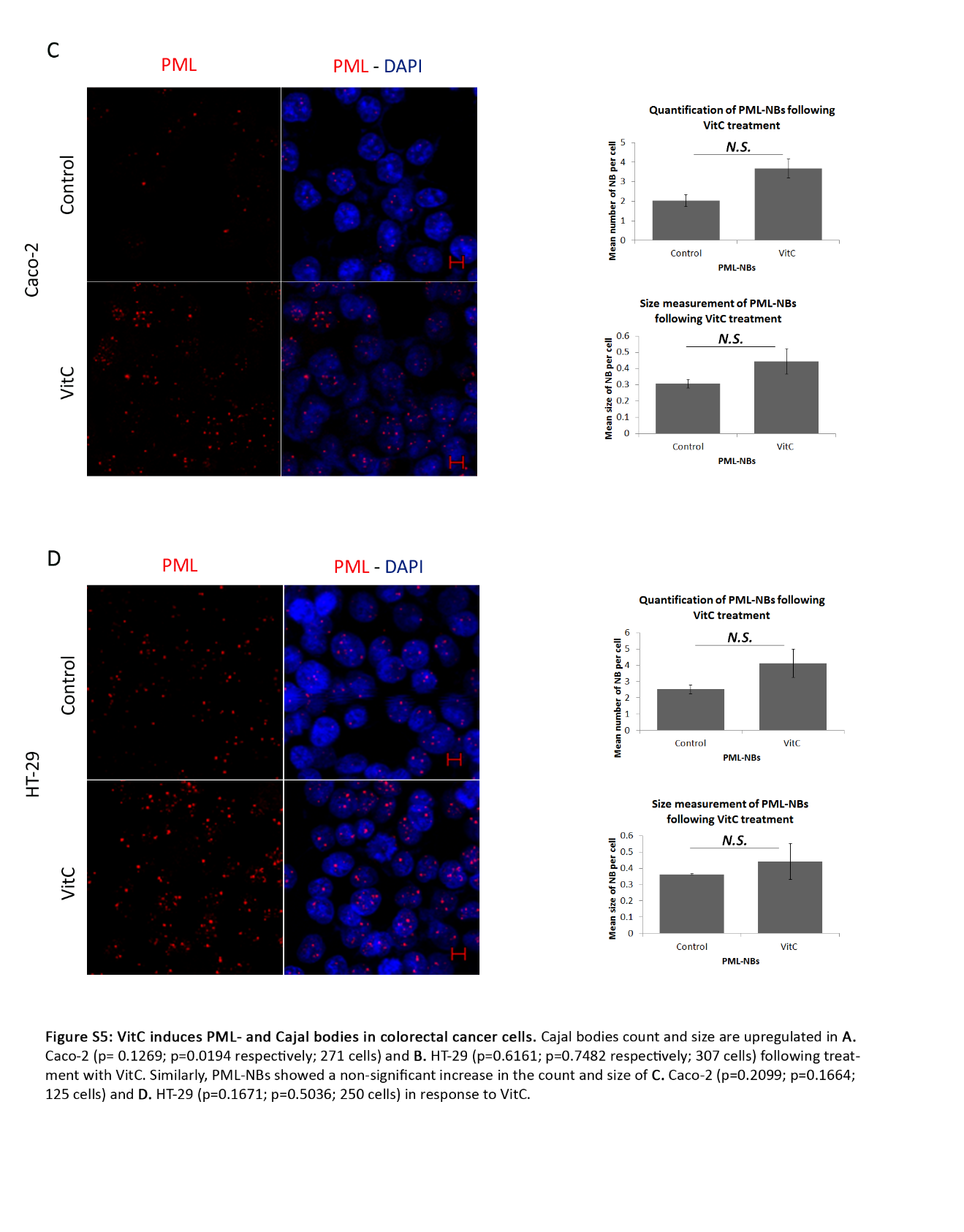


**Figure S5: VitC induces PML- and Cajal bodies in colorectal cancer cells.** Cajal bodies count and size are upregulated in **A.** Caco-2 (p= 0.1269; p=0.0194 respectively; 271 cells) and **B.** HT-29 (p=0.6161; p=0.7482 respectively; 307 cells) following treatment with VitC. Similarly, PML-NBs showed a non-significant increase in the count and size of **C.** Caco-2 (p=0.2099; p=0.1664; 125 cells) and **D.** HT-29 (p=0.1671; p=0.5036; 250 cells) in response to VitC.

**
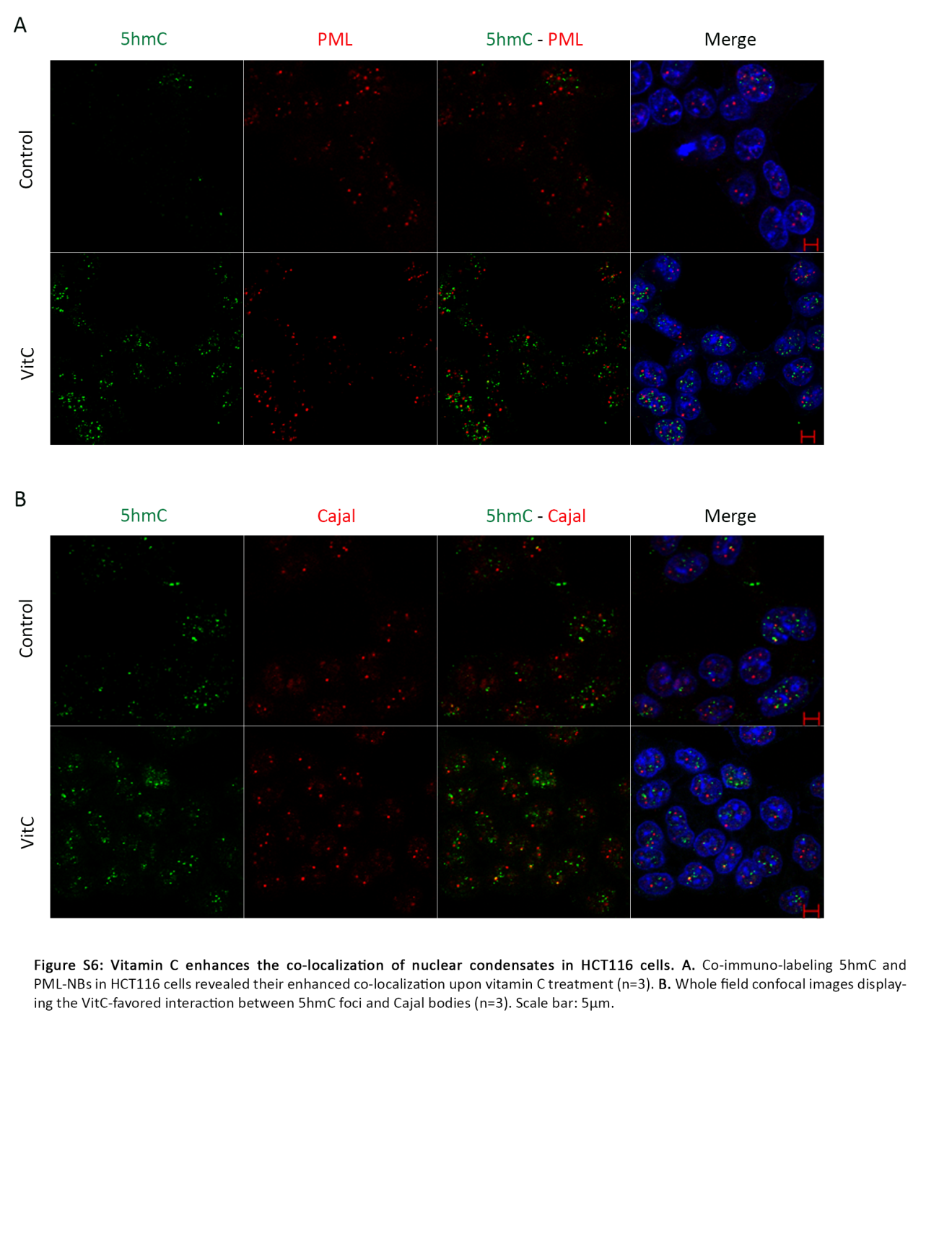
**

**Figure S6: Vitamin C enhances the co-localization of nuclear condensates in HCT116 cells. A.** Co-immuno-labeling 5hmC and PML-NBs in HCT116 cells revealed their enhanced co-localization upon vitamin C treatment (n=3). **B.** Whole field confocal images displaying the VitC-favored interaction between 5hmC foci and Cajal bodies (n=3). Scale bar: 5µm.


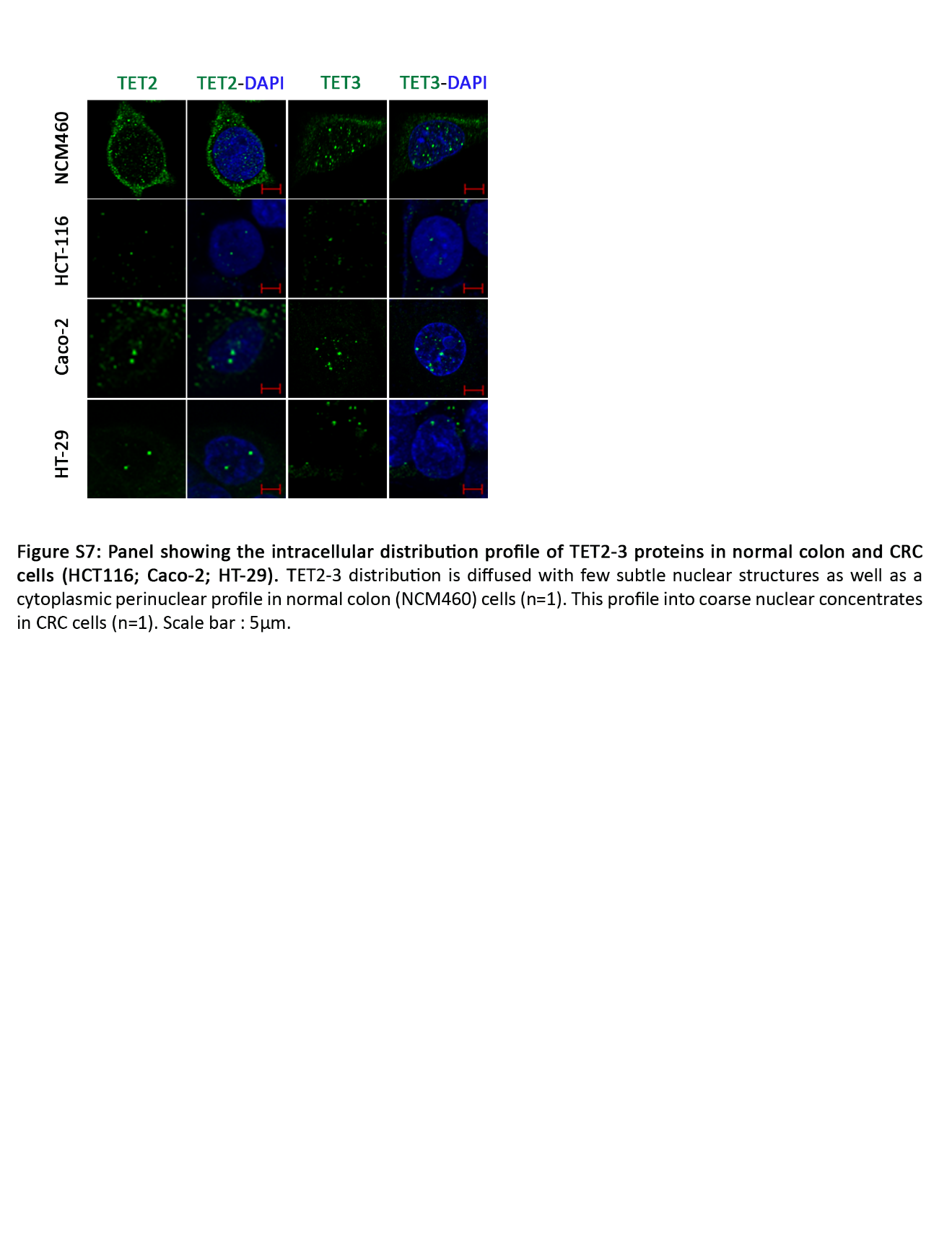


**Figure S7: Panel showing the intracellular distribution profile of TET2-3 proteins in normal colon and CRC cells (HCT116; Caco-2; HT-29).** TET2-3 distribution is diffused with few subtle nuclear structures as well as a cytoplasmic perinuclear profile in normal colon (NCM460) cells (n=1). This profile into coarse nuclear concentrates in CRC cells (n=1). Scale bar: 5µm.

Table S1: Co-localization percentage of the interdependent interaction between TET1/5hmC and Cajal bodies in HCT116 cells.

| **Percentage of Co-localization** | **Untreated cells** | **VitC-treated cells** |
| --- | --- | --- |
| %TET1-NBs and CBs positive cells out of total TET1 -NBs co-localized with CB | 15.89% ~ 16% | Not assessed |
| % TET1-NBs and CBs positive cells out of total Cajal bodies | 11.592% ~12% |  |
| %5hmC-foci and CBs positive cells out of total CBs | 11.22% ~ 11% | 19.633% ~ 20% |
| %CBs and 5hmC-foci positive cells out of total 5hmC foci | 12.418% ~ 12% | 8.939% ~ 9% |
